# Intraneural Lipoma: A Rare Cause of Median Nerve Compression

**DOI:** 10.7759/cureus.40074

**Published:** 2023-06-07

**Authors:** Mohammad O Boushnak, Mohamad K Moussa, Ali H Alayane, Antonia Gkotsi, Wissam El Kazzi

**Affiliations:** 1 Orthopedic Surgery, Université Libre de Bruxelles (ULB) Erasme University Hospital, Brussels, BEL; 2 Orthopedic Surgery, Clinique du Sport, Paris 5, Paris, FRA; 3 Orthopedic Surgery, Centre Orthopédique Santy, Lyon, FRA; 4 Orthopedic Surgery, Université Lbre de Bruxelles (ULB) Erasme University Hospital, Brussels, BEL

**Keywords:** nerve tumor, orthopaedic hand surgery, carpal tunnel syndome, median nerve entrapment, intraneural lipoma

## Abstract

Intraneural lipomatous tumors are rare lesions that mostly affect the upper extremities. These slowly growing tumors can have a serious neurological and functional impact when they reach a significantly large size. We report herein a case of a 53-year-old female who presented with a large median nerve intraneural lipomatous tumor causing compression-related signs. She was treated with monoblock excision of the tumor that was completely residing between the median nerve fibers. At her last follow-up, no median nerve deficits were recorded, and the patient went to full resolution.

## Introduction

Intraneural lipomatous tumor is a rare tumor that occurs mostly in the upper extremities. Although it may affect any nerve in the upper extremities, the median nerve is the most commonly involved [1,2]. It has been suggested that the incidence of this tumor is the highest in the fourth and fifth decades, with a female predominance [1,2]. Clinical presentation can be variable, ranging from neurological symptoms due to mass effect to cosmetic issues caused by the tumor’s large size [3-5]. Intraneural lipomas arise from the adipose cells that lie under a nerve’s epineurium [6]. Herein, we present a case of huge intraneural lipoma of the median nerve, and clearly describe how to approach these types of tumors.

## Case presentation

**Figure 1:**
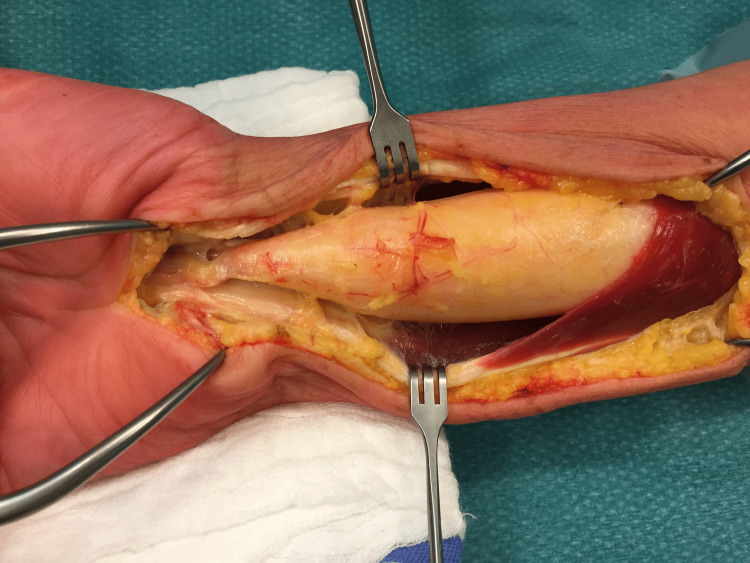
Intraoperative image showing the tumor lying inside the neural sheath of the median nerve.

A 53-year-old female patient presented to our hand clinic with a two-year history of an enlarging mass in her right forearm. Recently, she had visited her family doctor who referred her to our clinic. On physical examination, we noted tight skin on her forearm and an obvious palpable mass extending from the middle to the distal forearm. Neurological examination was almost unremarkable. Focusing on the median nerve, there was no alteration in the sensory propagation as attested by the negativity of Tinel’s sign, and by Dukran’s and Phalen’s tests. The thenar eminence was near normal in comparison to the contralateral hand with no signs of atrophy; in addition, thumb opposition was normal. The only remarkable finding was weakness in the flexion of both the interphalangeal joint of the thumb and the distal interphalangeal joint of the index finger. An electromyogram (EMG) was performed and showed anterior interosseus nerve compression and alteration of motor conduction of the median nerve at the level of the forearm. Due to her claustrophobia, magnetic resonance imaging (MRI) was aborted in the middle of the procedure. Eventually, we resorted to ultrasonography (US) to further delineate the mass. Ultrasound imaging showed a well-encapsulated lipomatous tumor with benign features adhering to the median nerve with dimensions of 116 x 30 x 35 mm. Consequently, surgical treatment with excisional biopsy was planned. Under general anesthesia and tourniquet control, a longitudinal incision was done from the middle of the forearm extending to the level of the carpal tunnel. The tumor was found to be lying inside the epineurium of the median nerve (Figure 1).

**Figure 2:**
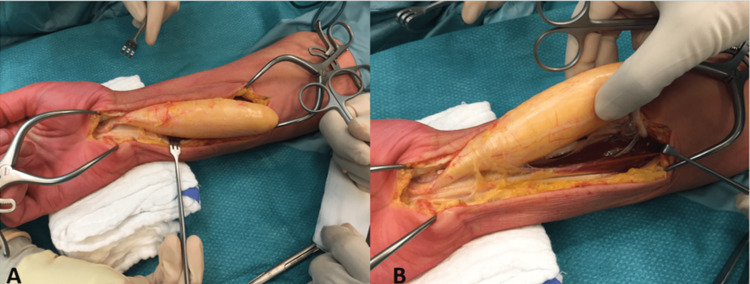
(A, B) Intraoperative image during dissection of the tumor of the median nerve sparing and protecting each individual fascicle.

The epineurium was incised, and the tumor was dissected carefully sparing the fascicles of the median nerve (Figure 2).

**Figure 3:**
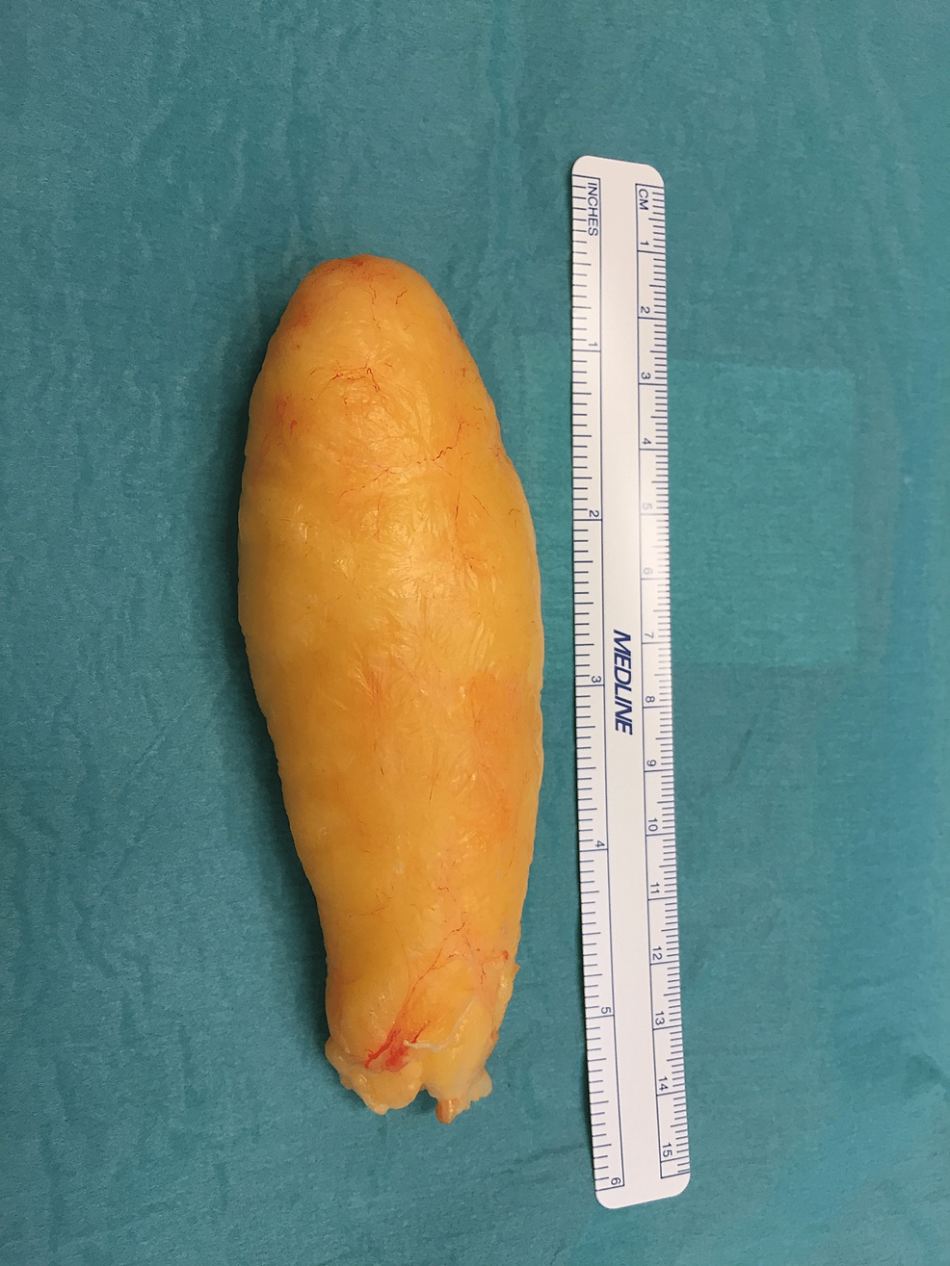
Intraoperative image showing the tumor after mono-bloc excision.

**Figure 4:**
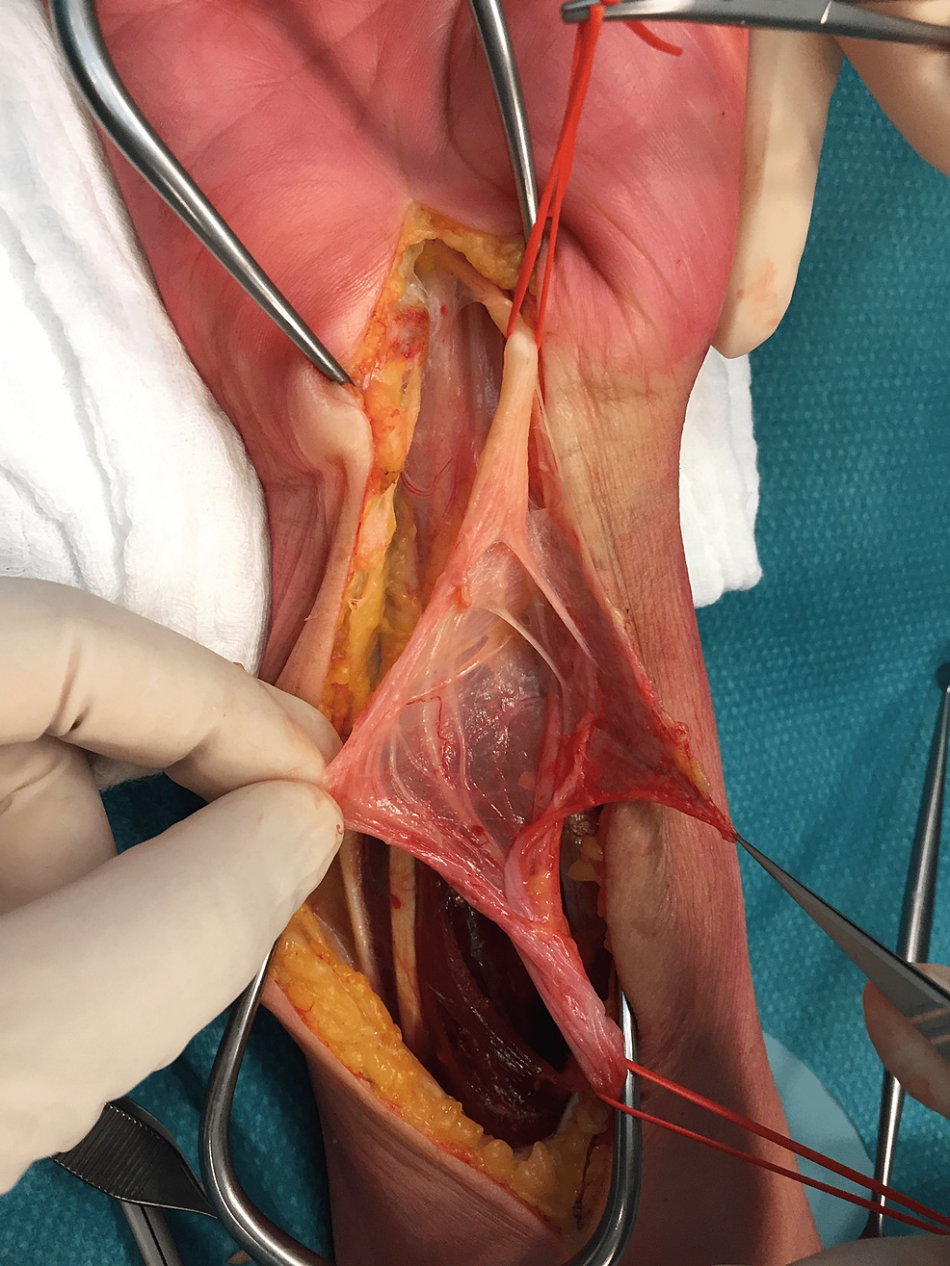
Intraoperative image showing the intact median nerve and its individual fascicles after mono-bloc excision of the tumor.

A mono bloc excision of a 120 x 50 x 25 mm lipomatous tumor was successfully carried out without injuring the fibers of the median nerve (Figures 3, 4).

**Figure 5:**
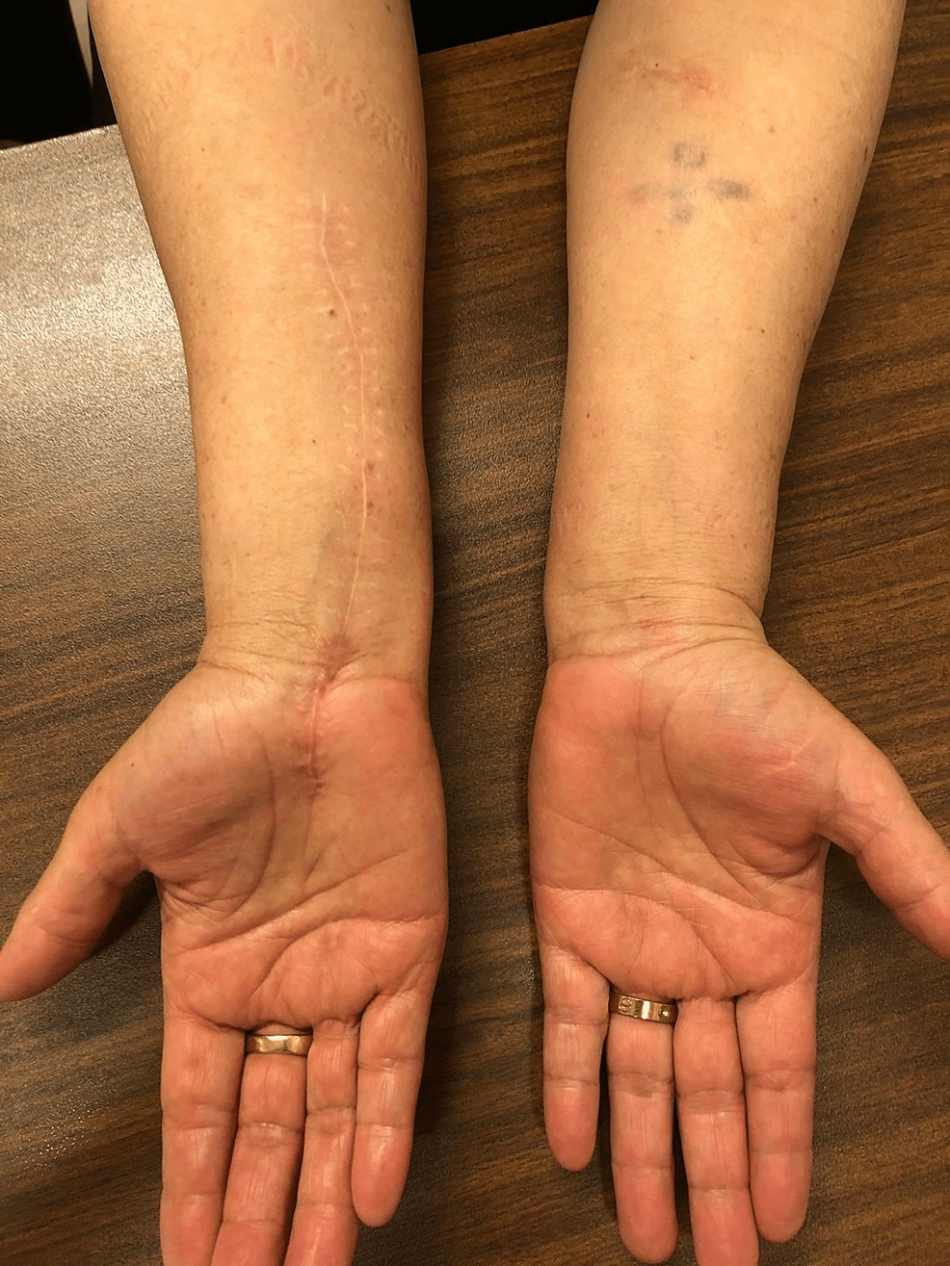
Image taken three years post-operation displays a well-healed scar and no signs of thenar eminence atrophy, as compared to the contralateral side.

Opening of the flexor retinaculum was obligatory due to the distal extension of the tumor to the proximal border of the carpal tunnel. The skin was then closed in a regular fashion and the wound healed without any complications. Pathological examination suggested a mature adipose tissue with a fine capsule confirming the diagnosis of intraneural lipoma. Postoperatively, the patient complained of numbness in the area innervated by the median nerve, which resolved completely after seven weeks. At three-year follow-up, the patient had complete thumb opposition when compared to the contralateral hand without any neurological symptoms or atrophy of the thenar eminence (Figure 5).

Complete recovery of the strength of thumb interphalangeal joint flexion and index distal interphalangeal joint flexion was observed, indicating complete neurological resolution of the anterior interosseous nerve.

## Discussion

Intraneural lipoma is a rare tumor that can affect peripheral nerves and the first reported case was in the year 1964 by Morley et al. [7]. When discussing intraneural lipomas, it is critical to clearly differentiate between intrinsic neuromas and extrinsic neuromas that cause neurological compromise. The former refers to tumors that arise under the epineurium of the nerve, whereas the latter refers to lipomas that occur outside the nervous tissue but can lead to neurological symptoms due to mass effect [8]. Three types of intrinsic lipomas have been described in the literature: encapsulated or intraneural lipoma, lipofibromatous hamartoma, and macrodystrophia lipomatosa [1]. Intraneural lipomas, or “true intraneural lipomas” as defined by Ruskoet et al., are well-encapsulated fatty tumors that arise from the adipose tissue of the epineurium of the nerve [9]. They do not usually invade the neural elements and can be clearly distinguished from the nervous tissue [9]. The second type is the lipo-fibromatous hamartoma which was described for the first time in 1953 [10]. It was found that this pathology is associated with childhood macrodactyly and growing forearm masses that progress with aging [2,6]. Lipofibromatous hamartomas have an unfavorable prognosis since the nerves will be invaded by adipose tissue [4]. The third type of intrinsic lipomas is macrodystrophia lipomatosa, a congenital disorder associated with overgrowth of the fibro-adipose tissue throughout the affected extremity in a disproportionate manner, thereby leading to cosmetic and, to a lesser extent, functional problems [11].

The most common location for intraneural lipoma is the median nerve [2,12]. Nonetheless, several papers reported its occurrence in other peripheral nerves of the upper extremity, such as ulnar, radial, suprascapular, brachial plexus, supraclavicular and digital nerves [9,13]. On the other hand, intraneural lipomas were also recorded in the lower extremities affecting posterior tibial, common peroneal, sciatic, and superficial peroneal nerves [14,15]. Thus, any upper or lower extremity nerve may be affected.

The clinical presentation of intraneural lipomas of the median nerve can be variable. When occurring at the level of the wrist inside the carpal tunnel, the major presenting symptom is median nerve neuropathy. This is due to the narrow space inside the carpal tunnel which does not allow expansion of the soft tissue, thereby leading to median nerve compression and to a clinical scenario that resembles carpal tunnel syndrome [12]. When occurring at the level of the forearm, rarely do intraneural lipomas present with compressive neuropathy. On the contrary, the main motives for medical advice are psychological, cosmetic, and localized pain at the level of the tumor [1]. Only a few cases in the literature have been identified in which the intraneural lipoma was in the forearm and patients had median nerve neuropathy, as in the case we presented [16].

MRI is the gold standard for diagnosing intraneural lipomas. It usually shows a sharply demarcated homogenous mass that has high intensity on both T1 and T2-weighted images. After erasing the fat signal, this mass will be hypointense on T1 and T2 images. Intraneural lipomas can be differentiated from other tumors with fibrous components by the lack of enhancement with intravenous gadolinium injections [17]. According to Capelastegui et al., MRI has a 96% positive predictive value in achieving the correct diagnosis when compared to histopathological findings [18]. US can also be a useful tool in diagnosing intraneural tumors and identifying their clear anatomy. The only drawback of the US is being operator dependent. Haldeman et al. anticipated that shortly high-resolution US will not only identify the nerve and the pathology but will also become a tool that can clearly describe intraneural anatomy [5].

The usual treatment for intraneural lipoma of the median nerve is surgical resection. Sometimes it is necessary to dissect between the fascicles of the nerve in order to completely excise the tumor [12]. Surgical excision of intraneural lipoma carries several risks and complications, including infection, scar sensitivity, nerve damage, complex regional pain syndrome, and contractures [19]. Sympathetic reflex dystrophy was also documented by Flores et al. when resecting a median nerve lipoma [20].

## Conclusions

In conclusion, it is indispensable for orthopedic surgeons to differentiate between the different types of intraneural adipose cell tumors. Surgical excision typically resolves simple intraneural lipomas, but it may result in neural compromise for more invasive types.
